# Use of an Abbreviated Geriatric Screening Tool in the Assessment of Older Cancer Patients’ Functional Status, Dependency, and Comorbidities: Cross-Sectional Audit and Observations From a Regional Cancer Center in Australia

**DOI:** 10.2196/16408

**Published:** 2020-04-07

**Authors:** Mathew George, Alexandra Smith

**Affiliations:** 1 North West Cancer Centre Tamworth Hospital Hunter New England Local Health District Tamworth Australia; 2 School of Rural Medicine University of New England Armidale Australia

**Keywords:** geriatric assessment, cancer, elderly, medical oncology, Australia

## Abstract

**Background:**

Malignancies are the leading cause of disease burden in Australia, comprising 19% of total diseases. Approximately 1 in 4 men and 1 in 6 women die from malignancies by 85 years of age, with patients aged 65 years and older contributing to 58% of diagnoses and 76% of cancer mortality. In the context of malignancy-related disease and age-related degeneration, there is a need for comprehensive assessment of older patients to plan for appropriate management and predict prognosis. The utility of available comprehensive geriatric assessment tools has been limited in routine practice because of their time-consuming nature, despite their informing clearer understanding of patients’ functional status, better clinical decision making, prevention of unpredictable admissions and emergency department overload, and support services planning. Though there are several promising tools available, there is a lack of literature on tools that can comprehensively assess functional status in an expedited fashion.

**Objective:**

This study aimed to document functional status and comorbidities among a geriatric oncology patient cohort attending a regionally located, dedicated cancer care facility, using the completed Adelaide tool assessments. This study documents cohort characteristics, including sociodemographics, malignancy type, and comorbidities. Secondarily, we observed the utility of an abridged functional assessment in the multidisciplinary team (MDT) management of older cancer patients.

**Methods:**

The study comprised a facility-based cross-sectional audit of results obtained from a screening tool administered to patients aged 65 years and older and attending an outpatient medical oncology clinic for management of cancer from late 2015 to 2017. Data relating to five domains were collected, including instrumental activities of daily living, activities of daily living, performance status, unintended weight loss, and exhaustion. Sociodemographic and disease-related factors were summarized as frequencies with percentages or mean with SD. Distribution of functional status based on sociodemographic characteristics, living status, disease-related factors, and comorbidities was analyzed using a chi-square test. Cumulative dependencies in the five domains were identified, and patients were classified as fit, vulnerable, or frail. Supplementary review of presentation notes for cases discussed at MDT meetings was undertaken to identify discrepancies.

**Results:**

A majority of the study population showed poor functional status, with 88.7% (243/274) categorized as vulnerable and 8.4% (23/274) as frail. Exhaustion and unintended weight loss were identified as the most common contributors to dependency. Polypharmacy was strongly associated with decreased functional status.

**Conclusions:**

The outcomes of this study are congruent with the existence of dependency in various domains, and with similar research in geriatric oncology. The Adelaide tool provided a useful basis for MDT discussion and management, where cases were referred to the MDT. We recommend further examination of the tool’s utility and impact in clinical decision making, and the distribution of dependencies in a rural cohort compared with metropolitan patients.

## Introduction

### Background

Globally, there are 962 million people aged 60 years or older, comprising 13% of the total population [1]. Advancements in medical sciences have led to an overall increase in life expectancy. According to the United Nations projections, the elderly population (>60 years) is estimated to reach 2.1 billion in 2050, or 22% of the projected world population [2]. The increase in life span is accompanied by challenges such as degenerative disorders, malnutrition, age-related disabilities, and increased risk of malignancies. Apart from physical illnesses, the elderly are also more vulnerable to social isolation, cognitive dysfunction, and emotional lability, with social isolation itself representing a significant risk factor for chronic noncommunicable conditions [3]. All these factors can culminate in a poor quality of life, and this recognition has catalyzed a strong global focus on the concept of healthy aging.

As per the recent Australian Burden of Disease Study (2011), malignancies are reported to be the leading cause of disease burden comprising 19% of total diseases. It has been estimated that 1 in 4 men and 1 in 6 women die because of malignancies by the age of 85, with patients aged 65 years and above contributing to more than half (58%) of diagnosed cases and three-quarters (76%) of cancer mortality in Australia [4]. Moreover, medical oncologists face the dual challenges of treating both the inherent problems associated with degenerative changes of aging and the malignancy-related disease itself [5]. As a result, there is a growing need identified among health care providers to access and use comprehensive geriatric assessments (CGAs) of patients to plan for the appropriate management and to predict prognosis [6]. The currently available CGA tools incorporate several domains such as nutrition, emotional wellbeing, cognition, social support, history of falls and injuries, comorbidities, polypharmacy, and disabilities [7-9]. However, although validated assessment is shown to have objective advantages over clinician judgment alone [10,11], the utility of these CGA tools and CGA-driven interventions has been limited in routine practice because of their time-consuming nature and consequent issues of completion rates and accuracy [12-14]. Hence, there is an imperative for many oncologists and multidisciplinary team (MDT) members to have access to an abridged tool to enable a clear understanding of elderly patients’ functional status, thereby informing better clinical decision making [15,16]. A comprehensive understanding of functional status among the elderly can also facilitate the prevention of unpredictable admissions and overload in the emergency department. Further, it points to the need for and can inform the planning of required support services and bed occupancy [17]. Though there are several promising tools available in practice, there is a relative lack of literature on clinical tools that can comprehensively assess functional status and particularly those that do so in an expedited fashion [18].

### Study Objectives

In this context, this study was designed to utilize a specific, abbreviated geriatric assessment (Adelaide tool) to document functional status and comorbidities among a geriatric oncology patient cohort attending a regionally located, dedicated cancer care facility. This was undertaken using existing patient- or proxy-completed Adelaide tool assessments. This study documents specific aspects of this cohort on the basis of sociodemographic characteristics, nature of malignancy, and coexisting morbidities, with the related aim of identifying patient characteristics that are associated with lower functional status scores. A secondary aim was to observe the utility of an abridged functional assessment in the MDT management of older cancer patients.

## Methods

### Study Design

This is a facility-based cross-sectional audit of results obtained from a screening tool administered among elderly patients aged 65 years or older who attended an outpatient medical oncology clinic for management and treatment of cancer.

### Study Setting

This study was conducted in a dedicated cancer care facility located in a large regional center in Australia, providing medical oncology, radiation oncology, and hematology services. The facility is a part of a wider health district encompassing numerous smaller centers and communities. The study site is situated over 400 km road or air travel from the closest capital city, and almost 300 km from the nearest large metropolitan center. The cancer care service also operates a number of regular oncology clinics in small rural communities within the wider health district, and services a geographical area of around 106,000 km^2^. The majority of patients from within the broader region and who are diagnosed with cancer are referred to this center for ongoing management and treatment if appropriate.

In the timeframe during which this study was conducted, patient visits and other interactions in the medical oncology and hematology areas of the center totaled around 26,000 inclusive of consultations, treatment visits, home nurse visits, and telephone follow-ups and telehealth appointments. The number of individual patients attending the center or its outreach clinics totaled 1255 in this period, with 689 (54.90%) of this cohort aged 65 years or older.

Of the 1255 patients attending the center in the study period, 557 (44.38%) presented to the medical oncology unit for a clinical consult. Of these 557 medical oncology patients, 350 (63.8%) patients were aged 65 or older, an increase in the proportion of older patients in the center overall and on the national estimates provided above—new medical oncology patients aged 65 years or older presenting in this period comprised 197 patients, or 56.3% (197/350) of all individual patients aged 65 years or older who attended the medical oncology clinic in the study timeframe.

In the wider hospital within which the oncology facility is situated, management of cancer and its treatment is provided by an MDT comprised of medical oncologists, radiation oncologists, hematologists, general and specialist surgeons involved in cancer-related procedures, pathologists, dieticians, social workers, and other allied health professionals who provide social, psychological, and nutritional support. The MDT meets fortnightly, and cases identified by clinicians on the basis of screening and assessment via methods such as the Adelaide tool or other means of selection by surgeons or other specialists are discussed. Recommendations regarding treatment, ongoing management, and/or any required referrals to allied health and support services such as community/home care are then determined and communicated to patients and their primary health care providers (eg, general practitioner). This approach aligns with current practice in the multidisciplinary management of oncology patients and incorporates recognition of geriatric assessment in such discussions and decision making [19,20].

### Recruitment

This study included patients aged 65 years and over, diagnosed with any type of malignancy, and attending the study center during the period from November 2015 to November 2017. Each new patient aged 65 years or over and attending the medical oncology clinic during the reference period was invited to complete the Adelaide tool, a screening questionnaire for the assessment of older people with cancer. Existing patients aged ≥65 years and attending the medical oncology clinic, and who were identified for possible referral to an MDT meeting, were also invited to complete an assessment in most instances where they had not done so previously.

### Study Tool and Data Collection

Initially, all new geriatric patients and some existing patients enrolled for cancer management were administered the screening questionnaire called the Adelaide tool screening questionnaire for the assessment of older people with cancer [21]. The Adelaide tool was developed by the Royal Adelaide Hospital Care Centre (Department of Health, South Australia) as a means of providing an abbreviated option for assessment of geriatric patients in clinical environments, and where time may not allow for initial extended and/or comprehensive assessments in all cases. A preliminary assessment of the validity of the Adelaide tool has been reported elsewhere [21].

Clinicians are able to use the tool to assess details related to self-rated health, medications use, memory, history of falls, hearing or vision impairment, activities of daily living (ADLs), instrumental activities of daily living (IADLs), social support, distress, pain, performance status, emotional wellbeing, and exhaustion. From this assessment, the Adelaide tool is used to classify functional status as fit, vulnerable, or frail.

This tool includes, in particular, the assessment of functional dependency in the following five domains referenced by other related studies [21]: (1) IADLs, (2) ADLs, (3) performance status (Karnofsky), (4) unintended weight loss, and (5) exhaustion [22,23].

Each new patient completed the Adelaide tool once, during an initial consultation at the medical oncology clinic before the commencement of treatment (where the treatment occurred). When required, assistance to complete the assessment was provided by an attending caregiver, friend, family member, or oncology nurse. In the small number of cases (n<5) where an assessment was mistakenly completed again at a later time, the later assessment was excluded from the analysis. On the basis of an examination of available records, all new patients (n=197) presenting to medical oncology in 2016 and 2017 were provided with a questionnaire and completed the questionnaire or were supported to do so as noted above. In addition, 77 existing patients who presented during this period and who had not previously completed the Adelaide tool also completed an assessment. Hence, a total of 274 assessments were completed and analyzed for this study.

In general, those patients who scored medium or high (vulnerable or frail) in the Adelaide tool were referred for consideration and discussion by the MDT. Owing to the retrospective nature of this study, however, this referral process to MDT was not always consistent. Therefore, on the basis of other factors, a patient who was classified as *fit* may have been referred for MDT discussion for other reasons. A patient who was classified as *vulnerable* may have been referred directly for treatment because of a number of factors, rather than referred to the MDT.

The data used in this study were therefore drawn from the Adelaide tool used for the screening of geriatric oncology patients (≥65 years), with the completed tool collected by an oncology nurse and maintained in the patient’s clinical records. The screening results were also presented in around half the cases at the regular MDT meetings within the cancer center, at which—as specified above—discussions of patients take place to inform recommendations for treatments and other clinical decision making. A brief supplementary review of presentation notes for those cases discussed at MDT meetings was undertaken to confirm that those patients discussed at MDT meetings had completed an Adelaide tool assessment. This review also aimed to identify any significant discrepancies, such as obvious misclassification of functional status in MDT presentations. Such discrepancies were not identified in any case.

The Adelaide tool screening data collected during the period mentioned above were accessed, collated, and entered by a research assistant. All information was deidentified.

### Statistical Analysis

Data were entered in Microsoft Excel with structured coding and analyzed using IBM SPSS, version 17.0. Patient sociodemographic and disease-related factors were summarized either as frequencies with percentages or mean with standard deviation. On the basis of the Adelaide tool, cumulative dependencies in the specified five domains were identified. For this purpose, the Katz index of independence in ADLs, Lawton IADLs scale, the Karnofsky performance status (KPS), weight loss more than 5%, and exhaustion score were considered [22-25]. The ADL two-item scale (2 without help and 1 with help/completely unable to do) was used as reported by Katz et al [24]. Similarly, to assess IADL in/dependence, items relating to the ability to use the telephone, go out, do shopping, and handle money and medications were considered. If the person was not able to perform any activity in the IADL related activity, they were considered dependent in that domain.

A similar approach was used to classify dependency for ADL-related activities. The KPS was assessed using eight coded responses. Appropriate percentages for each response were identified from the standard tool. Unintended weight loss of more than 5% in the last 6 months was also considered as a factor of concern. The exhaustion score was taken as a factor of concern if the person felt that everything they did was an effort or if they could not get going for a moderate amount or most of the time [25]. Patients’ functional status was classified as fit, vulnerable, or frail as per the categories reported by To et al [21]. Out of the five domains mentioned above, if there was no dependency in any domain, they were considered *fit*. Dependencies up to three factors were considered *vulnerable,* and 4 to 5 factors were deemed to be *frail*. Distribution of functional status on the basis of sociodemographic characteristics, living status, disease-related factors, and comorbidity status was analyzed using a chi-square test. Pain scores and distress scores across the three functional groupings were compared using the Kruskal Wallis (one-way analysis of variance) hypothesis test.

### Data Exclusion

Patients who had incomplete data were excluded from the study. If any patient had missing data in any one of the five domains (ADL, IADL, performance status, exhaustion score, and weight loss), they were still included in the final analysis on the basis of the contribution to the final classification on functional status. As an example, a patient who is functional in three domains, nonfunctional in one domain, and missing data in one domain will fall into the vulnerable status category, irrespective of their functional status in the missing domain. Hence, such patients were not excluded from the final assessment of functional status even though they had missing information in one domain.

### Research Ethics

This audit of screening tool data was approved by the local health district’s Research Ethics and Governance Office as a non-research activity comprising a retrospective cross-sectional audit and analysis of an existing patient screening tool dataset. Patient names and all other identifying data were removed from the database.

## Results

### Demographic and Clinical Characteristics: Overview

A total of 274 patients were included in this study, representing all new patients aged 65 years or older who presented to the medical oncology facility in the study period, plus the additional existing patients noted above. Of the 274 patients, 110 (40.1%) had been subjected to the MDT assessment, and the rest (164/274, 59.9%) had undergone only Adelaide tool assessment. All patients ≥65 years whose cases were presented at an MDT meeting had completed an Adelaide tool assessment. Owing to the retrospective nature of the study, reasons for nonresponse were not comprehensively documented.

The demographic and clinical characteristics of patients are summarized in Table 1. The mean age of patients was 75.4 years (SD 7.0 years). A total of 52.2% (143/274) of patients were males, and 12.0% (33/274) were living alone. Distribution of patient-related factors among those who were discussed in MDT and those who were administered the Adelaide tool alone were not found to be significantly different. The authors of this study have retained the separation of the two participant cohorts in the below tables predominantly for ease of representing the data as they were collected and to link the data with later discussion of the degree to which referral of patients to MDT discussions might be useful in the management of those patients.

Among men, the most common site of cancer was colorectal cancer, followed by prostate cancer. Among women, the most common site of cancer was breast, followed by lung. Of the 274 patients, 18 (65.7%) had stage 4 carcinoma, and 20 (7.3%) had a family history of cancer.

About 12.0% (33/274) of patients had more than four existing comorbidities, and 18.7% (52/274) of patients reported a history of at least one fall in the last 6 months. Of those patients who had completed the Adelaide tool alone, and had not been referred for discussion at an MDT meeting, 100.0% (164/164) reported four or fewer comorbidities. About half of the study population (125/274, 45.6%) reported unintended weight loss in the recent past (Table 1). Over two-thirds of the patients (241/274, 77.8%) reported at least one comorbidity. Ischemic and other cardiovascular diseases (159/274, 58.0%), hypertension (135/274, 49.1%), musculoskeletal disorders (135/274, 49.1%), gastrointestinal tract–related diseases (98/274, 35.7%), dyslipidemia (88/274, 32.1%), and diabetes (49/274, 17.8%) were the most common comorbidities identified among the patients.

**Table 1:** Demographic and clinical characteristics of geriatric patients attending a regional cancer care center (N=274).

Factors and categories		Adelaide tool + MDT^a^ (n=110)	Adelaide tool alone (n=164)
**Age (years)**			
	Mean (SD)	75.7 (7.3)	75.2(6.8)
	Median (IQR)	76 (69-81)	74(70-80)
**Sex, n (%)**			
	Male	59 (53.6)	84 (51.2)
	Female	51 (46.7)	80 (48.8)
**Living status, n (%)**			
	Living with spouse	69 (62.7)	16 (9.8)
	Living with children	7 (6.4)	100 (61.0)
	Living alone	23 (20.9)	10 (6.1)
	Living with others	11 (10.0)	38 (23.2)
**Site of cancer, n (%)**			
	Breast	17 (15.2)	16 (9.8)
	Colon or colorectal	22 (19.6)	23 (14.0)
	Pancreas, stomach, esophagus, or biliary tract	15 (13.5)	13 (7.9)
	Prostate	14 (12.5)	15 (9.1)
	Lung	13 (11.6)	15 (9.1)
	Female reproductive tract (uterus, ovary, or vagina)	7 (6.3)	6 (3.7)
	Liver metastasis	6 (5.4)	10 (6.1)
	Bone	3 (2.7)	1 (0.6)
	Head and neck	3 (2.7)	3 (1.8)
	Skin	2 (1.8)	2 (1.2)
	Brain metastasis	2 (1.8)	2 (1.2)
	Others	10 (8.9)	2 (1.2)
**Comorbidities, n (%)**			
	0 to 4	77 (70.0)	164 (100)
	More than 4	33 (30.0)	0 (0.0)
**Functional problems, n (%)**			
	Memory problems	24 (23.1)	36 (22.1)
	Vision problems (poor/blind)	101 (90.1)	156 (95.1)
	Hearing problems	99 (88.4)	147 (89.6)
	Weight loss	50 (50.5)	75 (47.2)
	Fall	22 (19.6)	30 (18.3)

^a^MDT: multidisciplinary team.

### Functional Status and Dependency in Functional Domains

Of the five functional domains included in the Adelaide tool, dependency because of exhaustion was the most commonly reported, followed by unintended weight loss. Dependency for household chores (31/274, 11.3%) and shopping (19/274, 6.9%) was found to be the maximum impaired IADL activity. Within the ADLs, continence (39/274, 14.3%) followed by bathing (24/274, 8.8%) were significantly impaired activities, making the elderly dependent for ADLs (see Table 2). Of 274 patients, 8 (2.9%) were identified as in the *fit* category of functional status, whereas a majority (243/274, 88.7%) belonged to the *vulnerable* status category (see Table 3).

**Table 2:** Distribution of dependency in various functional domains among geriatric patients attending a dedicated regional cancer care center (N=274).

Domain	Adelaide tool + MDT^a^ (n=110), n (%)	Adelaide tool only (n=164), n (%)	Total (N=274), n (%)
IADL^b^ dependent	16 (14.6)	24 (14.6)	40 (14.6)
ADL^c^ dependent	20 (18.2)	32 (19.5)	52 (18.9)
Karnofsky performance score <70%	37 (33.6)	71 (43.3)	108 (39.4)
Unintended weight loss >5%	50 (45.5)	75 (45.7)	125 (45.6)
Exhaustion	76 (69.1)	116 (70.7)	192 (70.1)

^a^MDT: multidisciplinary team.

^b^IADL: instrumental activities of daily living.

^c^ADL: activities of daily living.

Functional status was found to be similar across different demographic, clinical, and social support structure elements. Though there was an increased proportion of frailty among male patients, increased comorbidities, memory disturbance, a history of falls, and the smaller sample size could have precluded the result from attaining statistical significance. However, a larger number of medications (6 or more) was found to be significantly associated with frail functional status among elderly patients. Similarly, patients with frail functional status had higher pain or distress scores compared with patients with fit or vulnerable status (see Table 3).

**Table 3:** Distribution of functional status across sociodemographic and clinical characteristics (N=274).

Factors and categories		Fit (n=8)^a^	Vulnerable (n=243)^b^	Frail (n=23)^c^	*P* value^d^
**Age (years), n (%)**					**.20**
	<70	4 (3.0)	125 (92.6)	6 (4.4)	
	70 to 75	2 (3.2)	52 (83.9)	8 (12.9)	
	76 or more	2 (2.6)	66 (85.7)	9 (11.7)	
**Sex, n (%)**					**.56**
	Male	5 (3.5)	124 (86.7)	14 (9.8)	
	Female	3 (2.3)	119 (90.8)	9 (6.9)	
**Living status, n (%)**					**.30**
	Living with spouse	5 (3.0)	21 (91.1)	5 (5.9)	
	Living with children	0 (0.0)	154 (94.1)	10 (5.9)	
	Living alone	2 (3.1)	16 (85.1)	1 (11.5)	
	Living with others	1 (3.7)	52 (77.8)	7 (18.5)	
**Comorbidities, n (%)**					
	0 to 4	6 (2.5)	215 (89.2)	20 (8.3)	.30
	More than 4	2 (6.1)	28 (84.9)	3 (9.1)	.30
	Presence of memory disturbances	2 (3.3)	44 (73.3)	14 (23.3)	.30
	Fall >1 episode	1 (4.8)	16 (76.2)	4 (19.1)	.10
	IADL^e^ dependent	0 (0.0)	20 (50.0)	20 (50.0)	<.001
	ADL^f^ dependent	0 (0.0)	31 (59.6)	21 (40.4)	<.001
	KPS^g^ <70%	0 (0.0)	85 (78.7)	23 (21.3)	<.001
	Weight loss >5%	0 (0.0)	105 (84.0)	20 (16.0)	<.001
	Exhaustion	0 (0.0)	181 (94.3)	11 (5.7)	<.001
**Type of assessment^h^, n (%)**					**.60**
	Combined (Adelaide tool + MDT^i^)	3 (2.7)	100 (90.9)	7 (6.4)	
	Adelaide tool only	5 (3.1)	143 (87.2)	16 (9.8)	
**Number of medications, n (%)**					**.02**
	Fewer than 6	4 (1.9)	193 (91.5)	14 (6.6)	
	6 or more	4 (6.4)	50 (79.3)	9 (14.3)	
Distress score^j^, median (IQR)		3 (0-5)	3 (0-5)	6 (0-7)	.02
Pain score^j^, median (IQR)		3 (2-7)	5 (2-7)	5 (3-7)	.01

^a^2.9% (8/274).

^b^88.7% (243/274).

^c^8.4% (23/274).

^d^Chi-square test.

^e^IADL: instrumental activities of daily living.

^f^ADL: activities of daily living.

^g^KPS: Karnofsky performance status.

^h^For 7 patients–data on functional status are not available.

^i^MDT: multidisciplinary team.

^j^Kruskal-Wallis hypothesis test.

## Discussion

### Principal Findings

This study was carried out to assess the functional status of an elderly population diagnosed with malignancy and attending a dedicated cancer care center in a regional area using an abbreviated geriatric assessment tool (the Adelaide tool). It was found that a majority of the overall study population showed poor functional status, with 88.7% (243/274) of patients being categorized as having vulnerable functional status and 8.4% (23/274) as frail. Exhaustion and unintended weight loss were attributed as the highest contributors to dependency. Large proportions of this elderly population with malignancy were identified as having either vulnerable or frail functional status, and this reflects the existence of dependency in various domains.

Proportions of patients identified in this study with *fit* functional status (8/274, 2.9%) are less compared with the fit status (28%) reported in other studies involving the use of the Adelaide tool [21]. This could be explained by the differences in the distribution of types of cancers and a greater number of associated comorbidities in our study. However, the distribution of functional status across different sociodemographic and clinical factors was found to be similar in both the studies. Both studies identified the number of medications to have a positive association with poor functional status. Further, this study has also demonstrated the association of poor functional status with increased pain and distress scores. Considering that pain can directly limit various activities mentioned under IADL, ADL, and performance status, it is reasonable to assert that these attributes can lead to poor functional status.

An increased number of medications used was strongly associated with poor functional status in our study. This aligns with findings from other papers that show an association between polypharmacy in older patients with cancer and an increased risk of frailty and related complications [26-29]. It supports the importance of examining and discussing polypharmacy among older cancer patients as part of multidisciplinary oncology management, treatment decision making, and prognosis estimation [29-31].

Aside from the number of medications, however, several other factors such as the number of comorbidities, a history of one or more falls in the preceding 6 months, and memory loss associated with poor functional status did not achieve statistical significance. As this study aimed to identify the distribution of functional status among the study population, the small sample size addressing the primary objective may not have had enough statistical power to prove an association between these other factors and the functional status of older patients with cancer. Although it has been suggested in several studies that the associative and predictive value of many geriatric assessment domains is not always clear [32], there remains value in pursuing future research in regional, rural, and metropolitan cancer services to understand the utility of geriatric assessment domains in informing clinical decision making.

### Additional Observed Results

The study was not specifically designed to assess the ways in and the degree to which the tool was utilized in clinical decision making. However, it was observed during this study that the functional status assessment on the basis of the Adelaide tool was incorporated in the presentations made at MDT meetings (as evidenced by MDT presentation copies) and was therefore used in clinicians’ discussions around patient management and treatment decisions. In particular, the assessment informed clinicians’ discussions at MDT meetings regarding whether a patient required management, treatment, and support by members of a wider MDT or whether management by the individual medical oncologist was sufficient. Similarly, the determination of treatment method for patients, such as systemic therapy, concurrent chemotherapy-radiation therapy, or observation-only, may also be influenced by the baseline comprehensive functional status and its incorporation in clinician discussion of individual cases.

At least subjectively, therefore, clinicians appeared to find the Adelaide tool useful as a basis for assessing the immediate and ongoing need for multidisciplinary discussion and management of patients, with a specific view to considering functional status as it relates to treatments such as chemotherapy. Further research would be required to better examine the relationship between Adelaide tool results, MDT discussion, and clinician decisions, and to more precisely assess the tool’s relative ease of use for clinicians and patients. This supports the work and recommendations generated by recent research in relation to the use of geriatric assessment in the context of patient management and its potential impact on treatment decisions for older cancer patients [5,9,13,31].

### Limitations

Considering that the 5 domains are part of the overall functional status, categories of functional status (fit, vulnerable, and frail) tested across domains such as ADLs and IADLs could lead to incorporation bias. The findings of this study should be interpreted against the background of the following limitations. Several records had missing observation on patient characteristics. In this study, compared with other domains, dependency based on exhaustion was found among 70% of the included patients. This might have reduced the discriminative ability of the Adelaide tool to assess the different functional status.

In addition, as noted above, the small sample size in the study may not have achieved sufficient statistical power to fully address the primary objective of identifying the distribution of functional status and its association with other factors such as comorbidities.

Further, although clinicians intended to refer all those patients classified as vulnerable or frail for discussion at the MDT meeting, the analysis of the above available data highlights that this did not occur in all cases. The retrospective nature of this study itself presents a limitation in this respect, as the rationale for not referring vulnerable or frail patients for MDT discussion is not always clear. There is an opportunity here to further investigate and suggest potential improvements to the clinical process to optimize the utility of geriatric assessments in oncology patient management.

### Contribution

This study expands on recent work in developing and trialing an abbreviated CGA tool, namely the Adelaide tool [21], in the MDT management of older cancer patients. In doing so, it contributes to existing research and confirms a specific association between a greater number of medications and poorer functional status (*vulnerable* or *frail*) in older people with cancer. Further, it goes some way to confirming the utility of the Adelaide tool in the broader context of MDT approaches to the management of geriatric oncology patients, albeit with a small sample size mitigating further extrapolation of results.

### Conclusions

This study aimed to utilize the results of an abbreviated geriatric assessment tool to document functional status and comorbidities among a geriatric oncology patient cohort attending a regionally located, dedicated cancer care facility. This was undertaken using the existing Adelaide tool for geriatric patient assessments. This study documented specific aspects of this cohort, including sociodemographic characteristics, malignancy type, and comorbidities, and identified patient characteristics that are associated with lower functional status scores. In this patient cohort, it was found that a significant proportion of older patients were classified as vulnerable or frail. This outcome is congruent with the existence of dependency in various domains, and also reflects other research in the area of geriatric oncology and assessment. This has implications for future planning of oncology and related services in areas where there is a significant and increasing population aged 65 years and older.

Another secondary objective of the study was to examine the utility of an abridged functional assessment in the management of older cancer patients. The study confirmed the relative feasibility of integrating an abbreviated, comprehensive assessment of functional status in a clinical approach to the management of geriatric oncology patients, in particular using the Adelaide tool to achieve this. The functional status assessment on the basis of the Adelaide tool was used in clinicians’ discussions and related decision making, for example, regarding whether a patient required management, treatment, and support by members of a wider MDT or whether management by the individual medical oncologist was sufficient.

Besides clinical decision making, this tool could also be used to inform prognosis prediction and to assess the need for further supportive care, reflecting recent work in the area of geriatric assessment and its provision of greater insights into survival and related decision making regarding treatment options for older patients with cancer [33]. This would require more in-depth research with clinicians and a larger sample size.

This study, therefore, suggests that the Adelaide tool provides a useful basis for multidisciplinary discussion and management of older patients with cancer and that resultant information helps to form a snapshot of a local patient subpopulation and the distribution of dependencies and range of functional status. However, it is recommended that further research is undertaken to examine the tool’s impact on clinical decision making and MDT management of older cancer patients. Also, it is recommended that future attention be focused on the analysis of the distribution of dependencies in a rural cohort as compared with metropolitan patients, in addition to the incorporation of a larger sample size as means of extending the application of this work.

## Abbreviations

ADL: activities of daily living
CGA: comprehensive geriatric assessment
IADL: instrumental activities of daily living
KPS: Karnofsky performance status
MDT: multidisciplinary team
